# Novel Step Floating Islands VDMOS with Low Specific on-Resistance by TCAD Simulation

**DOI:** 10.3390/mi13040573

**Published:** 2022-04-04

**Authors:** Dongyan Zhao, Yubo Wang, Yanning Chen, Jin Shao, Zhen Fu, Baoxing Duan, Fang Liu, Xiuwei Li, Tenghao Li, Xin Yang, Mingzhe Li, Yintang Yang

**Affiliations:** 1 Beijing Engineering Research Center of High-Reliability IC with Power Industrial Grade, Beijing Smart-Chip Microelectronics Technology Co., Ltd., Beijing 102299, China; zhaodongyan@sgitg.sgcc.com.cn (D.Z.); wangyubo@sgitg.sgcc.com.cn (Y.W.); chenyanning@sgitg.sgcc.com.cn (Y.C.); shaojin@sgitg.sgcc.com.cn (J.S.); lixiuwei@sgitg.sgcc.com.cn (X.L.); litenghao@sgitg.sgcc.com.cn (T.L.); 2 Beijing Chip Identification Technology Co., Ltd., Beijing 102299, China; fuzhen@sgitg.sgcc.com.cn (Z.F.); liufang1@sgitg.sgcc.com.cn (F.L.); 3 Key Laboratory of the Ministry of Education for Wide Band-Gap Semiconductor Materials and Devices, School of Microelectronics, Xidian University, No. 2 South TaiBai Road, Xi’an 710071, China; yangxin_xd@163.com (X.Y.); limingzhezs@126.com (M.L.); ytyang@xidian.edu.cn (Y.Y.)

**Keywords:** VDMOS, electric field, breakdown voltage, specific on-resistance

## Abstract

A novel VDMOS with Step Floating Islands VDMOS (S-FLI VDMOS) is proposed for the first time in this letter, in order to optimize the breakdown voltage (*BV*) and the specific on-resistance (*R*_on,sp_). The innovative terminal technology of Breakdown Point Transfer (BPT) is applied to S-FLI VDMOS, which transfers the breakdown point from the high electric field region to the low electric field region, and the S-FLI VDMOS structure uses multiple layers of charge compensation blocks to generate multiple electric field peaks in the drift region in order to optimize the electric field distribution. In the TCAD simulation, the *BV* of the proposed S-FLI VDMOS is improved to 326 V, which is higher than that of 281 V for the conventional Si VDMOS with the same drift region length of 15 μm, and the *R*_on,sp_ is reduced from 21.54 mΩ·cm^2^ for the conventional Si VDMOS to 7.77 mΩ·cm^2^ for the S-FLI VDMOS. Compared with the conventional Si VDMOS, the current density of the effective current conduction path is increased when the forward bias is applied to the proposed device.

## 1. Introduction

Vertical Double-diffusion Metal Oxide Semiconductor (VDMOS) is an important component in the field of power semiconductor devices and has been widely used due to its high switching speed, low loss, and high breakdown voltage (*BV*) [1]. However, the main problem with VDMOS power devices is that specific on-resistance (*R*_on,sp_) increases sharply with the increasing *BV*, which greatly limits the development and application of VDMOS power devices. In order to alleviate the contradictory relationship between the *R*_on,sp_ and *BV*, several new structures have been proposed to reduce the *R*_on,sp_ of the drift region, including superjunction (SJ) VDMOS, floating island MOSFET (FLIMOS) etc. [2,3,4,5,6,7,8,9,10]. However, the manufacturing process of SJ VDMOS is difficult and requires a strict charge balance. When the device is turned on, the P-type impurity compensation layer of SJ VDMOS occupies the conduction channel of the device [4].

**Figure 1:**
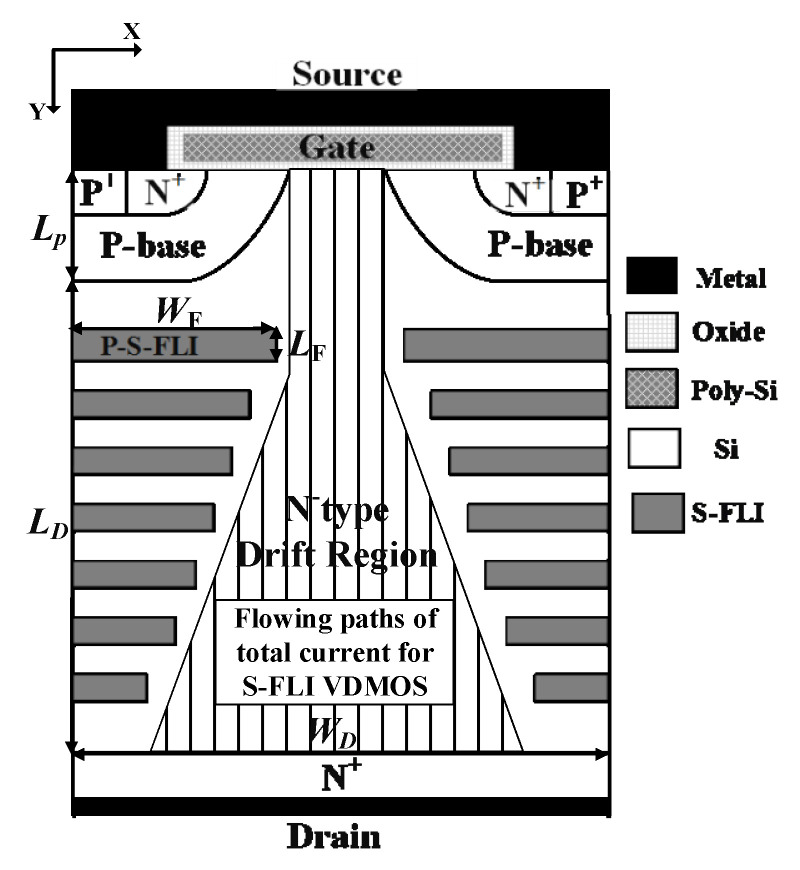
Cross section of S-FLI VDMOS with the number of rings of 7. Two Step Floating Islands at the same Y coordinate constitute a ring. (*L*_D_ is the length of N-drift region. *L*_P_ is the depth of the P-base. *W*_D_ is the width of device. *L*_F_ is the depth of Floating Islands. *W*_F_ is the length of Floating Islands. *N*_D_ is the concentration of N-drift region. *N*_SUB_ is the concentration of P-substrate. *N*_P_ is the concentration of P-base region. *Rings* is the number of rings, in which two Step Floating Islands at the same Y coordinate constitute a ring.).

In order to break the contradiction between the *R*_on,sp_ and *BV* of traditional Si devices, a novel VDMOS with Step Floating Islands VDMOS (S-FLI VDMOS) is proposed for the first time in this letter (seen Figure 1). The structure uses multiple layers of charge compensation blocks to generate multiple electric field peaks in the drift region, optimizing the electric field distribution. Compared with the conventional Si VDMOS, the *BV* is significantly improved, and the *R*_on,sp_ is effectively reduced. At the same time, the charge compensation region quickly shifts from the P-type base to the substrate, and when the forward bias is applied, the effective current conduction path of the device is increased. With the same *BV*, the *R*_on,sp_ of S-FLI VDMOS is smaller than that of FLIMOS [11,12,13,14]. The assisted depletion effect and electric field modulation can be applied in the lower-voltage devices to decrease the *R*_on,sp_ and higher-voltage devices to improve the *BV*, respectively.

## 2. Device Structure and Description

**Figure 2:**
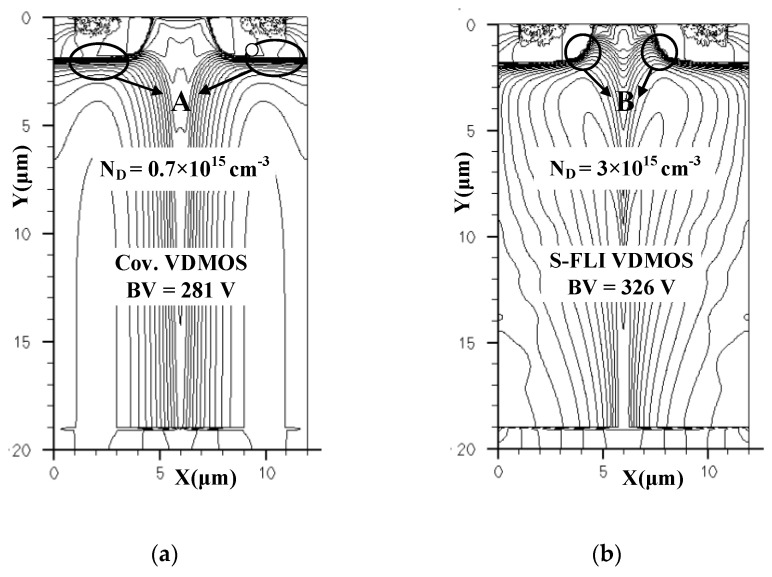
Distributions of the leakage current, (**a**) Cov.VDMOS, (**b**) S-FLI VDMOS.

The novel VDMOS with Step Floating Islands VDMOS (S-FLI VDMOS) is proposed. Figure 1 shows a cell of the proposed S-FLI VDMOS. The key feature is the Step Floating Islands [11,12,13,14] which consists of multiple P-type layers of charge compensation blocks in the N-type drift region. Two Step Floating Islands at the same Y coordinate in Figure 1 constituted a ring. The key steps of one feasible fabrication method for the proposed S-FLI VDMOS are shown below. First, the epitaxial growth N-Si Layer on the N^+^ Si Sub, and boron ions implantation and thermal diffusion form the P-type Floating Island. Next, the epitaxial growth N-Si Layer and boron ions implantation to form the second P-type Floating Island with a width slightly shorter than that of the first layer. This continues until seven Floating Islands are formed. Then, the thin gate oxide is employed with a typical thickness of 400 Å after a thermal growth process, and the source region is formed by phosphorus and boron ions implantation, respectively. Finally, a thick oxide passivation layer is deposited, and source, gate, and drain electrodes are formed. The electric field peaks are introduced to optimize the electric field distribution due to Step Floating Islands. Furthermore, S-FLI VDMOS exhibits better performance when the number of rings is increased. For the proposed S-FLI VDMOS, with the reverse drain voltage further increased, the electric field at area B (shown in Figure 2b) will reach the critical electric field of the Si material. The breakdown point will be transferred from area A (shown in Figure 2a) for the conventional Si VDMOS to area B for the S-FLI VDMOS by Breakdown Point Transfer (BPT).

The environment temperature is 300 K. *BV* is obtained at *V*_GS_ = 0 V, and *R*_on,sp_ is obtained at *V*_GS_ = 10 V. The physics models applied in the ISE simulation mainly includes Mobility (DopingDep High Field Sat Enormal), EffectiveIntrinsic Density (OldSlotboom), Recombination (SRH (DopingDep) and Auger Avalanche (Eparal)). The criterion of breakdown is BreakCriteria {Current (Contact = “drain” Absval = 1e−7)}. The breakdown condition is defined as the point at which the ionization integral equals unity. It is necessary to optimize the parameters in the numerical simulations. Some device parameters in the simulation are listed in Table 1. The simulation results based on the parameters in Table 1 are shown in Table 2.

## 3. Results and Discussion

**Figure 3:**
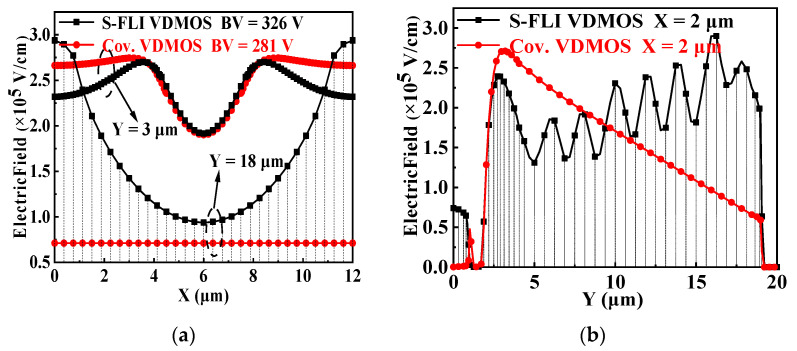
(**a**) Lateral electric field distributions for the conventional Si VDMOS and S-FLI VDMOS with *Rings* of 7: Y = 3 μm, Y = 18 μm and (**b**) Vertical electric field distributions for the conventional Si VDMOS and S-FLI VDMOS with *Rings* of 7: X = 2 μm.

The lateral electric fields and vertical electric fields distributions are shown in Figure 3a,b for the conventional Si VDMOS and S-FLI VDMOS, respectively. Figure 3a presents the lateral electric fields of the two devices at Y = 3 μm and Y = 18 μm. The electric field of the S-FLI VDMOS is higher compared with the conventional Si VDMOS, due to the multiple P-type layers of charge compensation blocks in the N-type drift region. However, the vertical electric fields of the conventional Si VDMOS and S-FLI VDMOS are different. For the conventional Si VDMOS, the highest electric field occurs at the junction of the P base and N-type drift region, resulting in a low *BV* of 281 V. For S-FLI VDMOS, as can be seen from Figure 3b, the peak electric field at the edge of drain has been introduced to the area B, and the vertical electric field distribution is extremely improved due to the electrical modulation of seven new electric field peaks introduced by Step Floating Islands. In addition, the auxiliary junctions created by multiple P-type layers of charge compensation blocks jointly sustain a high *BV* (326 V) for S-FLI VDMOS, which is improved by 16% compared to the conventional Si VDMOS (281 V) with the same *L*_D_.

**Figure 4:**
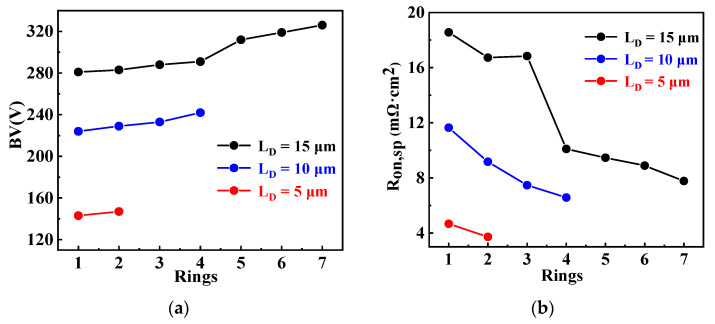
Dependences of (**a**) *BV* and (**b**) *R*_on,sp_ on *Rings* values and *L*_D_ for S-FLI VDMOS.

Figure 4 shows the dependence of *BV* and *R*_on,sp_ on *L*_D_ and the *Rings* for S-FLI VDMOS. It can be seen that the *BV* is improved and the *R*_on,sp_ is decreased with increasing *Rings*. This is because the new electric field peaks increased due to Step Floating Islands. This caused the *R*_on,sp_ to drop from 18.56 mΩ·cm^2^ to 7.77 mΩ·cm^2^, and the *BV* increased from 281 V to 326 V with the number of rings increased at the *L*_D_ of 15 μm.

**Figure 5:**
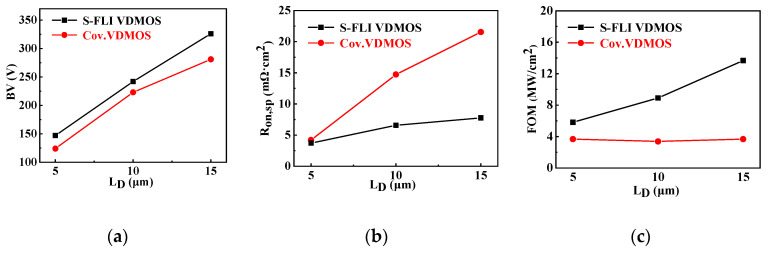
Dependences of (**a**) *BV*, (**b**) *R*_on,sp_ and (**c**) figure-of-merit (*FOM*) on *L*_D_ for the conventional Si VDMOS and S-FLI VDMOS.

The dependence of *BV*, *R*_on,sp_ and Figure-Of-Merit (*FOM* = *BV*^2^/*R*_on,sp_) on *L*_D_ for the conventional Si VDMOS and S-FLI VDMOS are shown in Figure 5. It is found that the *BV* of S-FLI VDMOS increases faster and saturates at a longer *L*_D_ as the *L*_D_ increases (*BV* > 300 V, at *L*_D_ =15 μm). Additionally, the *R*_on,sp_ of S-FLI VDMOS is lower than that of the conventional counterpart, yielding a higher *FOM* (13.68 MW/cm^2^) of S-FLI VDMOS than that (3.67 MW/cm^2^) of the conventional one with the same *L*_D_ of 15 μm.

**Figure 6:**
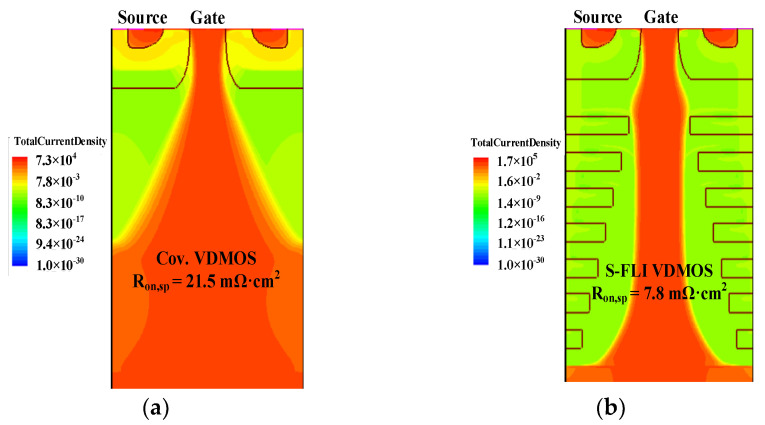
The distribution and flowing paths of total current for the (**a**) conventional Si VDMOS and (**b**) S-FLI VDMOS in on-state.

Figure 6 shows the distribution and flowing paths of total current for the conventional Si VDMOS and S-FLI VDMOS in on-state. Since the P-type S-FLI assists in depleting the N-type drift region in the off-state so that the optimum *N*_D_ is further increased, the current density for the proposed S-FLI VDMOS is higher compared to conventional Si VDMOS; thus, it is helpful for the *R*_on,sp_.

**Figure 7:**
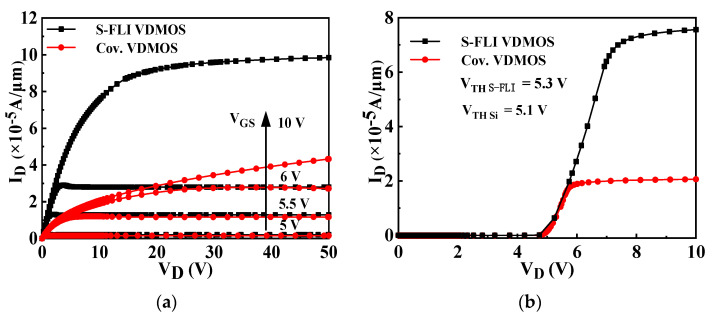
(**a**) Output characteristics and (**b**) transfer characteristics of the conventional Si VDMOS and S-FLI VDMOS.

The output characteristics and transfer characteristics of the conventional Si VDMOS and S-FLI VDMOS are shown in Figure 7. The threshold voltages V_TH_ of the two devices are approximately 5 V. At different gate voltages V_GS_ (5.0, 5.5, 6.0, 10 V), S-FLI VDMOS exhibits better output performance than the conventional counterpart, which leads to the result that the *R*_on,sp_ (7.77 mΩ·cm^2^) of S-FLI VDMOS is lower than that (21.54 mΩ·cm^2^) of the conventional VDMOS.

**Figure 8:**
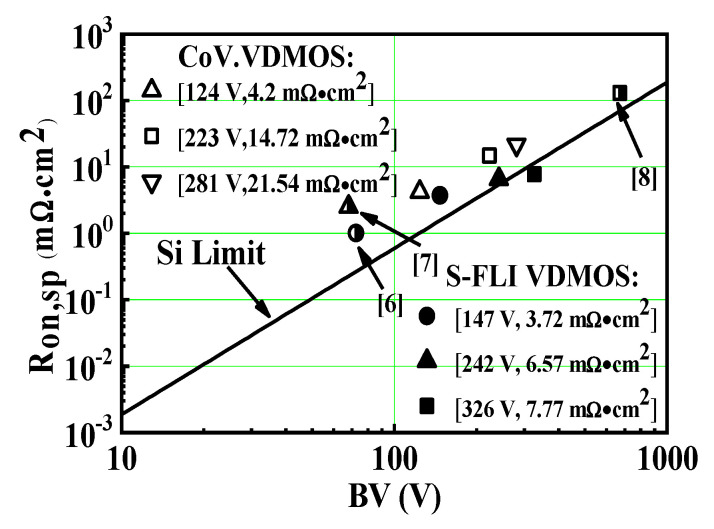
The *R*_on,sp_ versus *BV* with the ideal silicon limit, the reported structure and proposed Si VDMOS.

Figure 8 shows the *R*_on,sp_ versus *BV* for the S-FLI VDMOS, the conventional Si VDMOS, the reported structure, and the proposed VDMOS [6,7,8]. It can be seen that S-FLI VDMOS exhibits better performance at the *BV*, which further breaks the silicon limit under the optimized conditions.

## 4. Conclusions

The novel VDMOS with Step Floating Islands VDMOS (S-FLI VDMOS) is proposed in this letter. The structure uses multiple layers of charge compensation blocks to generate multiple electric field peaks in the drift region and optimize the electric field distribution. The *BV* (*BV* = 326 V) is significantly improved, and the *R*_on,sp_ (*R*_on,sp_ = 7.77 mΩ·cm^2^) is effectively reduced compared to the conventional Si VDMOS (*BV* = 281 V, *R*_on,sp_ = 21.54 mΩ·cm^2^) with the same *L*_D_ of 15 μm. When the forward bias is applied, the current density of the effective current conduction path of the S-FLI VDMOS is increased compared with the conventional Si VDMOS.

## Figures and Tables

**Table 1:** Device parameters in the simulation.

Device	Cov. VDMOS	SJ VDMOS	S-FLI VDMOS
***L*_D_ (μm)**	15	15	15
***Rings***	/	/	7
***N*_D_ (cm^−3^)**	0.7 × 10^15^	3.5 × 10^15^	3 × 10^15^
***N*_p_ (cm^−3^)**	5 × 10^17^	5 × 10^17^	5 × 10^17^
***N*_SUB_ (cm^−3^)**	1 × 10^14^	1 × 10^14^	1 × 10^14^

*L*_D_ is the length of N-drift region. *N*_D_ is the concentration of N-drift region. *N*_SUB_ is the concentration of P-substrate. *N*_P_ is the concentration of P-base region. *Rings* is the number of rings, in which a ring consists of two Step Floating Islands at the same Y coordinate.

**Table 2:** Simulation results for the Cov. VDMOS, SJ VDMOS and S-FLI VDMOS.

Device	Cov. VDMOS	SJ VDMOS	S-FLI VDMOS
***BV*(V)**	281	326	326
***R*_on,sp_** **(mΩ·cm^2^)**	21.54	6.93	7.77
***FOM*** **(MW/cm^2^)**	3.67	15.34	13.68

## Data Availability

Not applicable.
